# Harnessing electric potential: DLPFC tDCS induces widespread brain perfusion changes

**DOI:** 10.3389/fnsys.2013.00099

**Published:** 2013-11-28

**Authors:** Camilla L. Nord, Níall Lally, Caroline J. Charpentier

**Affiliations:** ^1^Department of Cognitive, Perceptual, and Brain Sciences, Institute of Cognitive Neuroscience, University College LondonLondon, UK; ^2^Department of Neurodegenerative Disease, Institute of Neurology, University CollegeLondon, UK; ^3^Experimental Therapeutics and Pathophysiology Branch, Intramural Research Program, National Institute of Mental Health, National Institutes of HealthBethesda, MD, USA; ^4^Division of Psychology and Language Sciences, Department of Cognitive, Perceptual and Brain Sciences, University College LondonLondon, UK

**Keywords:** arterial spin labelling (ASL), astrocytes, BOLD, brain stimulation, fMRI, glial cells, systems neuroscience, perfusion

Transcranial direct current stimulation (tDCS) is a noninvasive neuromodulatory technique with putative cognitive enhancing and therapeutic applications. Since the year 2000, almost 1000 papers have been published on tDCS, reflecting the possible significance of a cheap, safe, and easily applied neuromodulatory technology. Whether or not this potential is tapped depends on understanding how tDCS affects brain functioning, a question explored in a recent publication by Stagg et al. (2013); presently, its mechanism is largely unknown. Here, we discuss the implications of this recent research for understanding the effects of tDCS on neural processing.

tDCS is thought to decrease neuronal resting membrane potential beneath the anodal electrode by pumping in positive ions; vice versa for cathodal stimulation (Nitsche and Paulus, 2000). Systems-level human research has primarily used blood-oxygen-level-dependent (BOLD) contrast imaging to assess the underlying biological impact of tDCS (Keeser et al., 2011). The BOLD signal results from changes in the magnetic properties of hemoglobin, affecting local neurovascular coupling (Logothetis et al., 2001). Although BOLD depends on cerebral blood flow (CBF) changes, it is an indirect measure. By contrast, arterial spin labeling (ASL), a brain perfusion imaging technique, is a specific quantitative index of CBF (Petersen et al., 2006).

Stagg et al. studied the effect of tDCS on cerebral perfusion using ASL. Subjects (*N* = 12) underwent two tDCS sessions (one cathodal, one anodal) in a counterbalanced order, separated by a week. tDCS electrodes were applied to left DLPFC and the contralateral supraorbit. In each session, participants underwent a 40-min resting-state ASL functional MRI scan in which there was a 10-min baseline, 20-min of concurrent tDCS-ASL and a 10-min post-stimulation period. Separately, but using the same protocol, a second group of participants (*N* = 12) were scanned once under sham stimulation only. Stagg et al. compared resting-state CBF between these three periods and examined changes in functional connectivity between the regions beneath each electrode and the rest of the brain. The same analyses were conducted using the sham stimulation group to confirm that the changes were not driven by nonspecific temporal drifts.

Left DLPFC anodal tDCS resulted in increased perfusion to primary sensory and paracingulate cortices while cathodal stimulation evoked decreases in the thalami, in comparison with baseline. No significant difference between baseline and post-sham stimulation was found. Functional connectivity analyses revealed that anodal stimulation caused increased DLPFC coupling, but decreased coupling between left DLPFC and the thalami, brain stem, and cerebellum. Cathodal stimulation decreased coupling between left DLPFC and ipsilateral temporal, parietal, and occipital cortices. Functional connectivity analyses of the post-stimulation period revealed increased coupling between the left DLPFC and the primary sensorimotor cortices bilaterally after anodal tDCS relative to baseline. Widespread perfusion decreases occurred post-stimulation for both anodal and cathodal stimulation in comparison to the stimulation period.

These results are compelling as they describe widespread changes in blood flow occurring in regions outside of those being simulated both during and post-DLPFC stimulation, replicating and extending previous research. Both Lang et al. (2005) and Roche et al. (2011) demonstrated widespread perfusion changes following motor cortex stimulation in small sample between subject investigations. Stagg et al. (2013) however, assessed the ability of tDCS applied to DLPFC, a purportedly critical region in cognitive and clinical domains, and demonstrated distinct neural consequences during and following both anodal and cathodal stimulation in a counterbalanced within-subjects design. DLPFC is a frequent area of anodal stimulation for neuropsychiatric investigative treatment studies using tDCS.

Previous research suggests that tDCS after-effects have a non-synaptic mechanism of action, potentially involving changes in neural membrane function (Ardolino et al., 2005). This could bring about alterations in functional networks (Notturno et al., 2013), one interpretation of widespread perfusion changes post-stimulation. These changes are difficult to interpret, but bilateral DLPFC connectivity enhancements may be particularly beneficial for psychiatric disorders involving deficits in cognitive control.

However, two caveats should be mentioned. Although Stagg et al. demonstrate interesting effects, their analyses could be strengthened by direct comparison between the two groups. Statistically, to assess condition-specific between-group hypotheses, an analysis comparing group 1 (stimulation—baseline) to group 2 (stimulation—baseline) should be conducted; finding a statistically significant effect in one sample, but not another is not a valid approach to establish the between-group effect (Nieuwenhuis et al., 2011). Secondly, it is probable that the perfusion profile of tDCS effects change considerably during a cognitive task, since the behavioral effect of tDCS is task-specific (Leite et al., 2011), and enhanced when applied during a task (Andrews et al., 2011). Thus, it is unknown whether similar CBF alterations might occur following task-related stimulation. The results of Stagg et al. are nonetheless an interesting exploration of the resting-state perfusion effects of tDCS, which beckon neurophysiological underpinning investigations. It is currently unknown if tDCS induced changes arise from neuronal firing or from non-neuronal activity or an interaction between both.

The electrical current used in tDCS studies is insufficient to generate neuronal action potentials. Instead, spontaneous firing changes resulting from alterations in neuronal resting membrane potential are thought to underlie the neuromodulatory effects of tDCS (Bikson et al., 2004). Alternatively, its mechanism of action could involve the depolarization of astrocytic glial cells. Ruohonen and Karhu (2012) demonstrated computationally that tDCS may be sufficient to depolarize astrocytes. tDCS-evoked changes in neuronal plasticity could be secondary to changes in astrocytic activity. Astrocytes plays a prominent role in cerebral perfusion (Metea and Newman, 2006), which may explain the rapid CBF changes witnessed by Stagg et al. and others. This possibility has implications for clinical research in neurological and psychiatric disorders, where preliminary results have shown some clinical benefit of tDCS (Boggio et al., 2007; Kalu et al., 2012). Understanding physiological changes resulting from tDCS will be crucial to treatment development. Furthermore, any translational uses of tDCS will first require in-depth studies demonstrating clear and persistent effects of stimulation, which at present have not been shown.

While the clinical potential for tDCS is high, it is obscured by our lack of insight into its neural effects. Studies such as Stagg and colleagues' build on previous findings and pave the way to an improved understanding. Only with this comprehension can the full clinical possibility of tDCS be utilized and directed toward improving treatment.

## Author contributions

Camilla L. Nord, Níall Lally, and Caroline J. Charpentier wrote the manuscript together.
